# The effect of a smartphone-based coronary heart disease prevention (SBCHDP) programme on awareness and knowledge of CHD, stress, and cardiac-related lifestyle behaviours among the working population in Singapore: a pilot randomised controlled trial

**DOI:** 10.1186/s12955-017-0623-y

**Published:** 2017-03-14

**Authors:** Hui Zhang, Ying Jiang, Hoang D. Nguyen, Danny Chiang Choon Poo, Wenru Wang

**Affiliations:** 1 Alice Lee Centre for Nursing Studies, Yong Loo Lin School of Medicine, National University of Singapore, Clinical Research Centre, Block MD 11, Level 2, 10 Medical Drive, Singapore, 117597 Singapore; 2 Department of Information Systems, Computing School, National University of Singapore, Singapore, Singapore

**Keywords:** mHealth, Coronary heart disease, Health promotion, Primary prevention, Working population

## Abstract

**Background:**

Coronary heart disease (CHD) is the most prevalent type of cardiac disease among adults worldwide, including those in Singapore. Most of its risk factors, such as smoking, physical inactivity and high blood pressure, are preventable. mHealth has improved in the last decade, showing promising results in chronic disease prevention and health promotion worldwide. Our aim was to develop and examine the effect of a 4-week Smartphone-Based Coronary Heart Disease Prevention (SBCHDP) programme in improving awareness and knowledge of CHD, perceived stress as well as cardiac-related lifestyle behaviours in the working population of Singapore.

**Methods:**

The smartphone app “Care4Heart” was developed as the main component of the programme. App content was reviewed and validated by a panel of experts, including two cardiologists and two experienced cardiology-trained nurses. A pilot randomised controlled trial was conducted. Eighty working people were recruited and randomised to either the intervention group (*n* = 40) or the control group (*n* = 40). The intervention group underwent a 4-week SBCHDP programme, whereas the control group were offered health promotion websites only. The participants’ CHD knowledge, perceived stress and behavioural risk factors were measured at baseline and on the 4th week using the Heart Disease Fact Questionnaire-2, Perceived Stress Scale, and Behavioural Risk Factor Surveillance System.

**Results:**

After the SBCHDP programme, participants in the intervention group had a better awareness of CHD being the second leading cause of death in Singapore (*X* ^2^ *=* 6.486*, p* = 0.039), a better overall CHD knowledge level (*t* = 3.171, *p* = 0.002), and better behaviour concerning blood cholesterol control (*X* ^*2*^ = 4.54, *p* = 0.033) than participants in the control group.

**Conclusion:**

This pilot study partially confirmed the positive effects of the SBCHDP programme in improving awareness and knowledge of CHD among the working population. Due to the small sample size and short follow-up period, this study was underpowered to detect significant differences between groups. A full-scale longitudinal study is required in the future to confirm the effectiveness of the SBCHDP programme.

**Electronic supplementary material:**

The online version of this article (doi:10.1186/s12955-017-0623-y) contains supplementary material, which is available to authorized users.

## Background

The fact that coronary heart disease (CHD) is the most prevalent type of cardiovascular disease (CVD) among adults has become a major concern for public health [1]. It was estimated that eight million people died worldwide due to CHD in 2013 and this figure will continue to increase to 11.1 million by the year 2020 [2]. CHD is projected to remain the number one global killer in the year 2030 [3]. In Singapore, CHD has been the second leading cause of death since 2007, accounting for 15.5% of the total deaths in 2013 [4].

Work-related stress is often found to be associated with CHD [5–7]. In addition, the detrimental effect of CHD on the working population has caused productivity loss and economic burden [8]. Working adults with CHD exhibit poorer work performance, have lower income, and experience job loss because of disease-related diminished work capacity [9]. Premature death of workers caused by CHD is another dire consequence [10]. In Singapore, CHD places a heavy financial burden on working individuals. The cost of medical treatment for a single heart attack episode ranges from 6,000 to 40,000 Singapore dollars [11].

Although CHD affects many aspects of a person’s life, most of its risk factors, such as high blood pressure, abnormal lipid profile, smoking, and physical inactivity, are preventable [12]. Previous studies have reported that working adults lack adequate knowledge and awareness of CHD, and therefore, they were less able to recognise symptoms or adopt a healthy lifestyle to reduce their risks [13–17]. In Singapore, a national survey revealed that 60% of its adult residents exceeded the recommended daily calorie intake, and more than 54% reported that they have never exercised [18]. This calls for a need to develop innovative methods of primary prevention to help the working population prevent CHD.

With the rapid evolution of technology in the past decades, mobile health (mHealth), which includes both web-based and smartphone applications (apps), has been increasingly used in chronic disease management [19–22] and health promotion [23–27]. mHealth represents a convenient and accessible way for the public to improve their health and overall welfare. Several studies conducted in Western countries have demonstrated the effectiveness of mHealth in improving self-management skills for people with diabetes [28] and hypertension [29]. The focus in these studies is the use of smartphone technology for delivering self-management education. A recent systematic literature review conducted by Beatty et al. [30] found that three studies had used mobile phone technology for the delivery of cardiac rehabilitation and evaluated health outcomes in patients with CHD [31–33]. These studies supported the feasibility and acceptability of the use of mobile technology in health promotion and disease management.

In Singapore, increased smartphone use and a robust 4G network set the foundation for mHealth development [34, 35]. To the best of our knowledge, research has not yet been conducted in Singapore to evaluate the effectiveness of mHealth in improving awareness and knowledge of CHD among the working population. Therefore, this study was designed to examine the feasibility and efficacy of a newly developed 4-week smartphone-based coronary heart disease prevention (SBCHDP) programme in improving awareness and knowledge of CHD, perceived stress as well as cardiac-related lifestyle behaviours among the working population of Singapore.

## Methods

### Study design and participants

A pilot randomised controlled trial (RCT) was conducted in Singapore. Ethical approval was sought from the Internal Review Board of the National University of Singapore before the actual launch of the study (NUS-IRB reference code: B-15–059). The participants were recruited from various institutions or companies through a poster advertisement. Working individuals who were English speakers, had a full-time job, were aged 21–65 years old, and used smartphones were recruited. Those who had heart diseases, worked in a healthcare-related field (such as physicians, nurses, and pharmacists), and/or participated in other heart-related educational programmes were excluded. Billingham et al. [36] recommended that the sample size for a pilot study should be 36 per group. In addition, given that the estimated attrition rate is 10%, a sample size of 80 was used in the current pilot RCT.

### Intervention—the smartphone-based coronary heart disease prevention programme

**Fig. 1:**
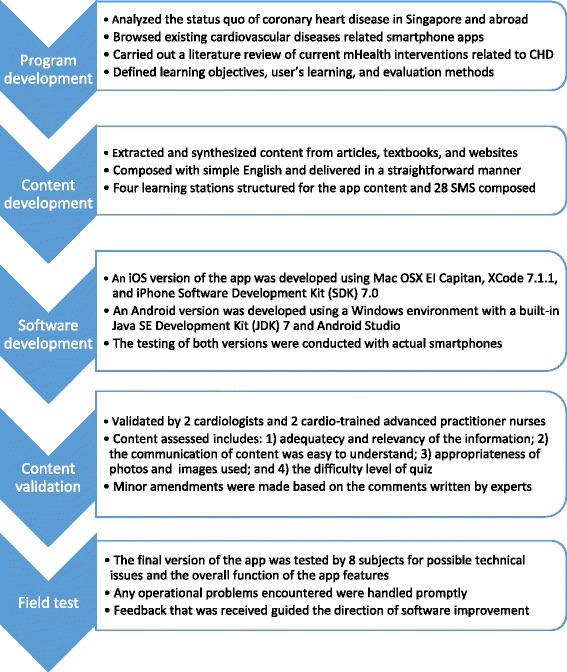
Development process for the SBCHDP program

**Fig. 2:**
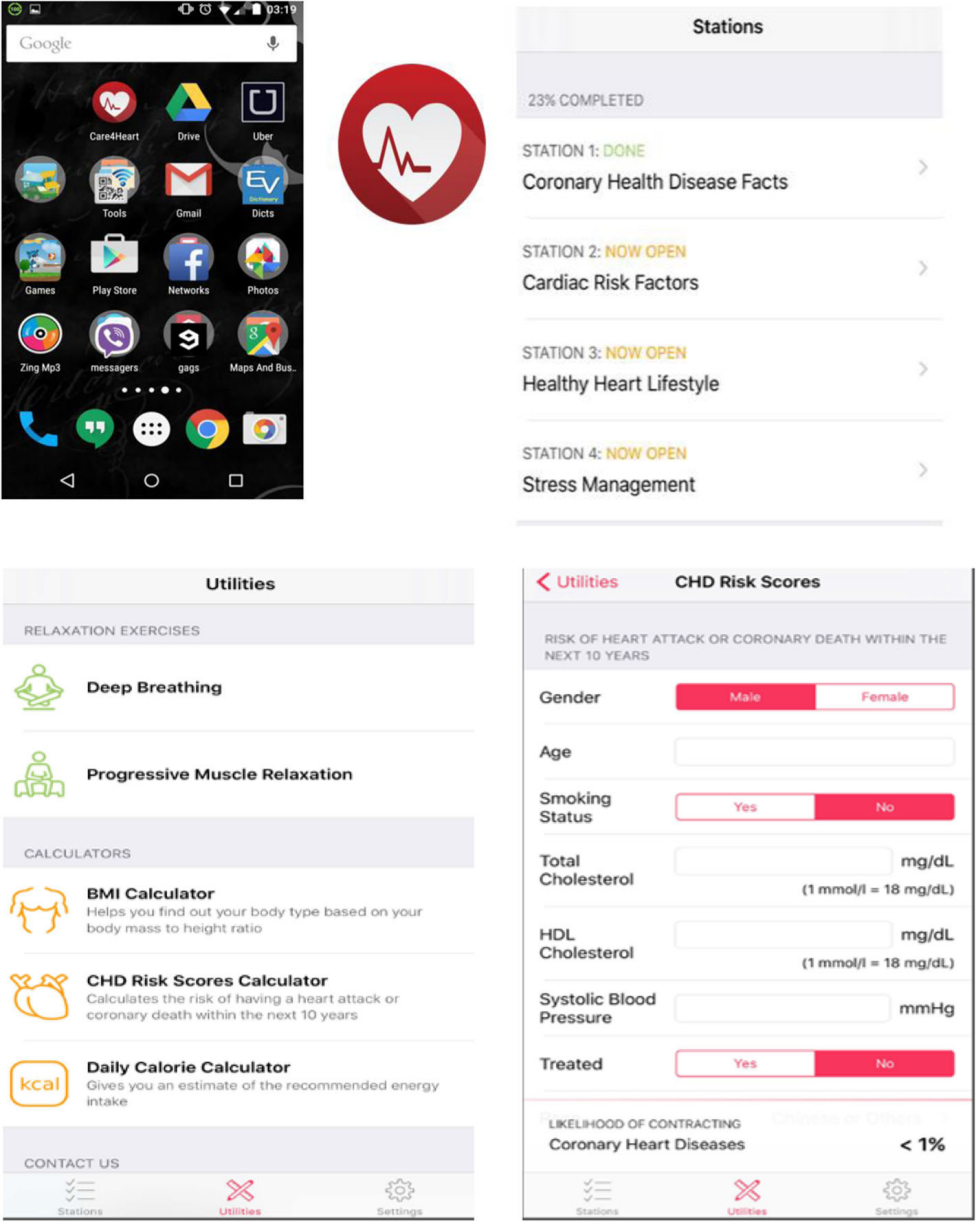
Care4Heart app icon and screenshots of app features

The 4-week SBCHDP programme was developed; it comprised a newly developed mobile app named Care4Heart, a 20-min briefing session, and a daily short message service (SMS). The Care4Heart app was specifically developed for working adults in Singapore. The development process of the Care4Heart app is summarised in Fig. 1, and the details have been published elsewhere [37]. There are four learning modules in the Care4Heart app for the participants to study in one month. The first learning module covers the physiology of the heart, the prevalence of CHD in Singapore, and the common signs and symptoms of CHD. The second module elaborates on the modifiable and non-modifiable cardiac risk factors. The third module provides information on healthy-heart lifestyles, including diet patterns, optimal check-up regimens, exercise, and smoking cessation. The last learning module focuses on stress management and the possible causes, signs, and symptoms of stress. On average, it takes approximately 10–20 min to read the content of each learning module, depending on the participant’s literacy. In addition, two demonstrative relaxation video clips (i.e., deep breathing exercise and progressive muscle relaxation) were included in the app. Three function icons were developed for body mass index (BMI) calculation, daily caloric-intake calculation with food pictures for reference, and 10-year CHD risk prediction [38]. Citations were provided in the app for reference and for information reliability. A technical helpdesk and contacts were available under the “settings” tab for technical support. The app icon and some app features are displayed in Fig. 2. Concurrently, a daily SMS offering healthy tips for CHD prevention was sent to the participants for a duration of four weeks.

The SBCHDP programme was offered to the participants of the intervention group, while the participants in the control group were provided the website addresses of the Singapore Heart Foundation (SHF) and the Health Promotion Board (HPB) for arbitrary exploration.

### Outcome measures

The primary outcomes of the study were the participants’ awareness and knowledge of CHD, and the secondary outcomes included the participants’ perceived stress level as well as their cardiac-related lifestyle behaviours.

### Awareness of CHD

The participants’ awareness of CHD was assessed by 3 multiple-choice questions, which were developed based on the information released by the Singapore Ministry of Health. These questions were developed to assess the participants’ awareness of CHD; specifically, the mortality rate of CHD, the gender risk factor, and the fact that CHD is the second leading cause of death in Singapore.

### Heart disease fact questionnaire-2

The participants’ knowledge of CHD was examined using the Heart Disease Fact Questionnaire-2 (HDFQ-2), which comprised 25 dichotomous questions (true or false answers). These questions assessed the participants’ knowledge of the modifiable and non-modifiable risk factors related to CHD, including age, gender, family history, blood glucose control, blood lipid control, blood pressure management, physical activity, smoking, and obesity. The total score ranged from 0 to 25, with higher scores indicating a better knowledge of CHD. The HDFQ-2 has been demonstrated to have a good internal consistency and test-retest reliability with a Cronbach’s alpha of 0.84 and Pearson's product-moment correlation coefficient of 0.89 [39].

### Perceived stress scale-10

The participants’ stress levels were measured using the Perceived Stress Scale-10 (PSS-10). The PSS-10 is a self-reported instrument designed to measure the level to which one appraises situations in his/her life as uncontrolled, unpredictable, and overwhelming [40]. It includes 10 items and uses a 5-point Likert scale to assess the perceived stress within the last month. The total score ranges from 0 to 40, with a higher score implying a higher level of stress. The PSS-10 has been reported to have a good internal consistency with a Cronbach’s alpha of greater than 0.80 [41].

### Behavioural risk factor surveillance system questionnaire

The participants’ cardiac-related lifestyle behaviours were assessed by 23 questions extracted from the 2013 version of the Behavioural Risk Factor Surveillance System (BRFSS) questionnaire [42]. The BRFSS was developed by the Centres for Disease Control and Prevention and is the most widely used questionnaire to assess risk behaviours that are associated with heart disease, including exercise, blood pressure and cholesterol control, body weight control, tobacco use, and alcohol consumption [42]. The BRFSS has been demonstrated to have good reliability and validity [43, 44]. The 23 questions that we used in this study are presented in the Additional file 1.

A self-developed survey was used to assess the users’ feedback of the SBCHDP programme. The participants’ socio-demographic data, including age, gender, marital status, occupation, ethnicity, monthly income, educational levels, current health condition, weight, height, and family history of CHD, were also collected.

### Data collection procedure

**Fig. 3:**
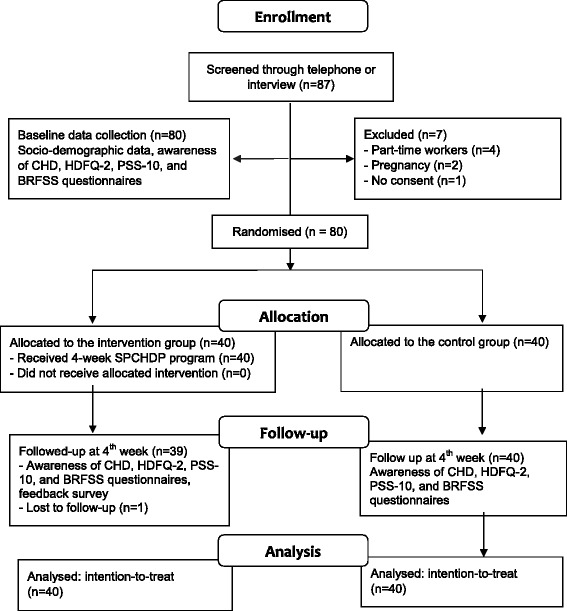
The CONSORT flow diagram for data collection. Legend: CHD: coronary heart disease; SPCHDP: smartphone-based coronary heart disease prevention; HDFQ-2: Heart Disease Fact Questionnaire-2; PSS-10: Perceived Stress Scale-10 (PSS-10); BRFSS: Behavioural Risk Factor Surveillance System

The data collection procedure is illustrated in Fig. 3. A total of 87 potential participants were identified and screened, of which 7 participants were excluded. Eligible participants were randomised to either the intervention group or the control group. The baseline data were immediately collected. For the participants in the intervention group, a researcher (HZ) conducted a 20-min individual briefing session to instruct them on how to download and use the Care4Heart app. The participants were also informed that a daily SMS that provides CHD prevention tips would be sent to their mobile phone for four weeks. For those allocated to the control group, the researcher advised them to browse health promotion information from the websites of the SHF and the HPB.

Data were collected at two time points: at baseline and immediately after the programme ended (four weeks from baseline). A total of 79 participants completed the second survey; one participant dropped out due to loss of contact. All participants were reimbursed S$20 for their participation in the study.

### Data analysis

Data were analysed using SPSS 23.0. The intention-to-treat (ITT) method was adopted for missing values, using the last observation carried forward imputation technique. The chi-square test was used to test the differences of awareness and lifestyle behaviours related to CHD between the two groups, while independent sample *t*-tests were used to test the differences in stress levels and total CHD knowledge between the two groups. Statistical significance was set at a *p*-value of less than 0.05.

## Results

Baseline data were acquired from a total of 80 working adults. The majority of the participants were Chinese (*n* = 61, 76.3%), female (*n* = 52, 65%), aged between 21 and 40 years old (*n* = 62, 77.5%), and at least college-educated (*n* = 62, 77.5%). More than half were married (*n* = 42, 52.5%) and had less than 10 years of working experience (*n* = 46, 57.6%). Nearly half of the participants (*n* = 35, 43.8%) worked in an information technology- or engineering-related field, 37.5% (*n* = 30) of the participants had other occupations, 15% (*n* = 12) of the participants worked in clerical services, and 3.8% (*n* = 3) of the participants worked in the teaching profession. There were no significant differences between the intervention and control groups in terms of the baseline socio-demographic data (Table 1).

**Table 1:** Demographic characteristics of intervention and control groups

Demographic characteristics	Intervention group (*n* = 40)		Control group (*n* = 40)		
	*n*	%	*n*	%	*x* ^2^ (*p*)
*Age group (years)*					4.868 (0.182)
21-30	20	50.0	14	35.0	
31-40	15	37.5	13	32.5	
41-50	2	5.0	4	10.0	
51-65	3	7.5	9	22.5	
*Gender*					.879 (0.348)
Male	12	30.0	16	40.0	
Female	28	70.0	24	60.0	
*Ethnicity*					1.886 (0.598)
Chinese	33	82.5	28	70.0	
Malay	5	12.5	9	22.5	
Indian	1	2.5	2	5.0	
Others^a^	1	2.5	1	2.5	
*Marital status*					3.208 (0.073)
Married	17	42.5	25	62.5	
Single	23	57.5	15	37.5	
*Education*					2.218 (0.528)
No formal education	1	2.5	0	0.0	
Secondary School	7	17.5	10	25.0	
ITE/Polytechnic/JC	16	40.0	12	30.0	
University	16	40.0	18	45.0	
*Monthly income (SGD)*					3.643 (0.303)
< 1500	2	5.0	4	10.0	
1500 -3499	31	77.5	24	60.0	
3500 -4999	6	15.0	8	20.0	
≥ 5000	1	2.5	4	10.0	
*Occupation*					0.724 (0.868)
Admin/Clerical	6	15.0	6	15.0	
IT/Engineering	19	47.5	16	40.0	
Teaching	1	2.5	2	5.0	
Others^b^	14	35.0	16	40.0	
*Years of working*					6.153 (0.188)
< 5	17	42.5	10	25.0	
5 -10	9	22.5	10	25.0	
11- 20	11	27.5	10	25.0	
21- 30	1	2.5	6	15.0	
31- 50	2	5.0	4	10.0	
*Existing chronic diseases*					8.398 (0.210)
Yes	3	7.5	9	22.5	
No	37	92.5	31	77.5	
*Family history of CHD*					1.920 (0.166)
Yes	1	2.5	4	10.0	
No	39	97.5	36	90.0	

^a^including Javanese, Burmese; ^b^including clerical support workers, service and sales workers, property agents, chefs, security guards, delivery attendants, event managers, drum trainers, construction researchers, research analysts, accountants, financial specialists, and lab attendants; *SGD* Singapore Dollar, *ITE* Institute Technology Education, *JC* Junior College, *IT* information technology, *CHD* coronary heart disease

Regarding the awareness of CHD, there was no significant difference between the intervention and control groups at baseline. There were significantly more participants in the intervention group who were aware that ‘*CHD is the second leading cause of death in Singapore*’ (*X* ^*2*^ = 6.486, *p* = 0.039) after the 4-week SBCHDP programme.

Table 2 shows the comparisons of CHD knowledge assessed using the HDFQ-2 between the intervention and control groups. At baseline, a significant difference was found in the total HDFQ-2 mean scores, with significantly better scores being reported by the participants in the control group (*t* = -2.118; *p* = 0.038). In contrast, the participants in the intervention group had significantly better scores than those in the control group (*t* = 3.171; *p* = 0.002) after the intervention. Specifically, there were significantly more participants in the intervention group who reported the right answers for the following four statements: (1) A family history of heart disease increases the risk of CHD (*X* ^2^ = 3.914, *p* = 0.048); (2) The risk of heart disease will be reduced if sugar levels are under control (*X* ^*2*^ = 5.165, *p* = 0.023); (3) People with diabetes tend to have low HDL cholesterol (*X* ^*2*^ = 7.366, *p* = 0.007); and (4) Diabetic people can reduce their risk of developing heart disease if they keep their weight under control (*X* ^2^ = 5.333, *p* = 0.021).

**Table 2:** Comparison of HDFQ-2 before and after the intervention between the intervention and control groups

Statements	Pre-test		*p*	Post-test		*p*
	Intervention group (*n* = 40)	Control group (*n* = 40)		Intervention group (*n* = 40)	Control group (*n* = 40)	
HDFQ-2 n (%)^b^						
1. Family history of heart disease increases the risk of HD.	34(85.0%)	35(87.5%)	0.745	39(97.5%)	34(85.0%)	0.048*
2. The older the person is, the greater the risk of having HD.	27(67.5%)	32(80.0%)	0.204	38(95.0%)	33(82.5%)	0.077
3. Smoking is a risk factor for HD.	39(97.5%)	39(97.5%)	1.000	40(100%)	39(97.5%)	0.314
4. Stopping smoking will lower the risk of HD.	36(90.0%)	37(92.5%)	0.692	37(92.5%)	37(92.5%)	1.000
5. High blood pressure is a risk factor for HD.	38(95.0%)	40(100%)	0.152	40(100%)	39(97.5%)	0.314
6. Keeping BP under control reduces risk for HD	38(95.0%)	40(100%)	0.152	40(100%)	40(100%)	^a^
7. High cholesterol is a risk factor for HD.	37(92.5%)	39(97.5%)	0.305	40(100%)	39(97.5%)	0.314
8. Eating fatty foods does not affect cholesterol levels.	37(92.5%)	37(92.5%)	1.000	35(87.5%)	34(85.0%)	0.745
9. High HDL puts you at the risk of HD.	35(87.5%)	37(92.5%)	0.456	38(95%)	37(92.5%)	0.644
10. High LDL puts you at risk for HD.	39(97.5%)	39(97.5%)	1.000	39(97.5%)	39(97.5%)	1.000
11. Being overweight increases the risk for HD.	40(100%)	40(100%)	^a^	40(100%)	39(97.5%)	0.314
12. Regular physical activity lowers the risk of HD.	27(67.5%)	33(82.5%)	0.121	38(95.0%)	40(100%)	0.152
13. Only exercising in gym or class lowers the risk of HD	33(82.5%)	36(90.0%)	0.330	36(90.0%)	38(95.0%)	0.396
14. Walking and gardening lower the risk of HD.	30(75.0%)	31(77.5%)	0.793	37(92.5%)	34(85.0%)	0.288
15. Diabetes is a risk factor for developing HD.	30(75.0%)	29(72.5%)	0.799	38(95.0%)	36(90.0%)	0.396
16. High blood sugar puts a strain on the heart.	31(77.5%)	33(82.5%)	0.576	38(95.0%)	34(85.0%)	0.136
17. High blood sugar increases cholesterol and the risk of HD.	28(70.0%)	31(77.5%)	0.446	36(90.0%)	34(85.0%)	0.499
18. The risk of HD will be reduced if sugar levels are under control.	30(75.0%)	33(82.5%)	0.412	38(95.0%)	31(77.5%)	0.023*
19. People with diabetes rarely have high cholesterol.	34(85.0%)	35(87.5%)	0.745	39(97.5%)	36(90.0%)	0.166
20. Diabetic people who keep their cholesterol under control will lower the risk of HD.	35(87.5%)	37(92.5%)	0.456	36(90.0%)	37(92.5%)	0.692
21. People with diabetes tend to have low HDL cholesterol.	16(40.0%)	17(42.5%)	0.820	29(72.5%)	17(42.5%)	0.007*
22. Diabetic people can reduce the risk of HD if they keep their blood pressure under control.	33(82.5%)	38(95%)	0.077	39(97.5%)	35(87.5%)	0.090
23. Diabetic people can reduce their risk of developing HD if they keep their weight under control.	34(85.0%)	37(92.5%)	0.288	40(100%)	35(87.5%)	0.021*
24. Men with diabetes have a higher risk of HD than women with diabetes	17(42.5%)	14(35%)	0.491	12(30.0%)	12(30.0%)	0.647
25. Women have a higher risk of HD after menopause	15(37.5%)	23(57.5%)	0.073	33(82.5%)	28(70.0%)	0.189
Total Mean (SD) ^c^	19.8 (3.0)	21.1 (2.1)	0.038*	22.9 (1.5)	21.4 (2.5)	0.002*

^a^: No statistic was computed because statement 6 and 11 is a constant. ^b^: Chi-square test; ^c^: Independent Sample *t*-test, *HD* heart disease, *HDL* high density lipoprotein, *LDL* low density lipoprotein, *BP* blood pressure, *SD* standard deviation; * Significance at *p* <0.05

Table 3 represents the comparisons of perceived stress levels and lifestyle behaviours related to CHD between the intervention and control groups. There were no significant differences at baseline between the two groups. After intervention, there were no significant differences in the perceived stress levels assessed using the PSS-10 between the two groups (*p* = 0.911). With regard to the BRFSS, there were no significant differences in the lifestyle behaviours between the two groups after intervention, with the exception of behaviours related to blood cholesterol control (*X* ^*2*^ = 4.54, *p* = 0.033). At follow-up, there was a significantly greater proportion of participants in the control group (*n* = 14, 37.8%) who reported that they were informed that their blood cholesterol was high by healthcare professionals compared to those in the intervention group (*n* = 5, 15.2%).

**Table 3:** Comparison of PSS-10 and BRFSS before and after the intervention between the two groups

Variables	Pre-test		*p*	Post-test		*p*
	Intervention group (*n* = 40)	Control group (*n* = 40)		Intervention group (*n* = 40)	Control group (*n* = 40)	
PSS-10 Mean (SD) ^a^	17.0 (5.3)	15.7 (5.2)	0.281	16.1 (4.8)	16.2 (5.1)	0.911
BRFSS n (%) ^b^						
General health (good)	35 (87.5%)	36 (90.0%)	0.645	35 (87.5%)	34 (85.0%)	0.879
History of diabetes (yes)	2 (5.0%)	1 (2.5.0%)	0.210	3 (7.5%)	2 (5.0%)	0.635
Medication (yes)	4 (10.0%)	5 (12.5%)	1.000	5(12.5%)	4 (10.0%)	0.723
Overweight	14 (35.0%)	16 (40.0%)	0.288	13 (32.5%)	17 (42.5%)	0.314
BP check (yes)	34 (85.0%)	34 (85.0%)	1.000	36 (90.0%)	36 (90.0%)	1.000
High BP (yes)	3 (7.5%)	7 (17.5%)	0.311	1 (2.5%)	3 (7.5%)	0.305
Exercise (yes)	25 (62.5%)	31 (77.5%)	0.222	24 (60.0%)	26 (65.0%)	0.644
Cholesterol check (yes)	33 (82.5%)	35 (87.5%)	0.755	33 (82.5%)	36 (90.0%)	0.330
High cholesterol (yes)	8 (24.2%)	14 (40.0%)	0.201	5 (15.2%)	14 (37.8%)	0.033*
Smoking (yes)	7 (17.5%)	2 (5.0%)	0.154	6 (15.0%)	2 (5.0%)	0.146
Alcohol drinking (yes)	11 (27.5%)	15 (37.5%)	0.557	13 (32.5%)	15(37.5%)	0.822

^a^:Independent Sample *t*-test; ^b^:Chi-square test; *BP* blood pressure; * Significance *p* < 0.05

The results of the feedback regarding the SBCHDP programme from the participants in the intervention group are summarised in Table 4. The majority of the participants agreed/strongly agreed that the SBCHDP programme improved their knowledge of CHD and that the newly developed Care4Heart app was easy to use and was convenient to access. In addition, the qualitative data from the open-ended question *“What ways can the app be improved based on your experience?*” indicated that the participants perceived that the SBCHDP programme was informative (e.g., “*good information app*”), feasible (e.g., “*app is just nice and convenient to use*”), and applicable (e.g., “*increasing the popularity of the app will benefit more people*”). The participants also commented that the areas to improve on included developing the app to be available in multiple languages, adding more pictures, and providing information regarding strategies for the management of heart disease.

**Table 4:** User’s feedback about the SBCHDP programme (*n* = 39)

Statement	Strongly disagree *n* (%)	Disagree *n* (%)	Not sure *n* (%)	Agree *n* (%)	Strongely agree *n* (%)
1. I feel that the intervention helped me improve my knowledge of CHD	0 (0.0%)	0 (0.0%)	4 (10.3%)	27 (69.2%)	8 (20.5%)
2. I find this app easy to use	0 (0.0%)	0 (0.0%)	4 (10.3%)	30 (76.9%)	5 (12.8%)
3. The app is convenient to access	0 (0.0%)	0 (0.0%)	2 (5.1%)	30 (76.9%)	7 (17.9%)
4. I feel that other people may benefit from this intervention	0 (0.0%)	0 (0.0%)	3 (7.7%)	26 (66.7%)	10 (25.6%)

## Discussion

This study is the first study conducted in Singapore to examine an mHealth approach to educating the working population about CHD prevention. The results from the current study revealed that, compared to the control group, there were more participants in the intervention group who were aware that CHD is the second leading cause of death in Singapore. This partly affirmed the feasibility of disseminating disease prevention messages to a wide audience by mHealth. However, awareness of gender risk and CHD mortality rate was not found to be significantly different between the two groups after the SBCHDP programme. In the current study, since the participants in the control group were provided with the website addresses of the SHF and the HPB, they had access to similar disease prevention information via those two websites.

In addition, while the Care4Heart mobile app and SMS intervention appeared to be an alternative method for delivering CHD educational content, websites perform a similar function and are also accessible from mobile devices. In this case, participants in the control group may have had increased access to the web-based content via their mobile devices. Overall, the sample size in the present pilot study may be underpowered for the detection of the intervention effect.

The results from the current study showed that the participants in both the intervention and control groups generally had good baseline knowledge regarding CHD risk factors, which was independent of the participants’ socio-demographic characteristics, such as educational level, age, ethnicity, marital status, occupation, medical history, or family history of heart diseases. While this was inconsistent with the findings of several previous studies [45], it may reflect the impact from the collaborative efforts of various governmental and non-governmental organisations in the prevention and control of CVD in Singapore. Many strategic health education campaigns and events that specifically target different populations have been launched and are implemented by the HPB at various locations, including workplaces, industries, and communities [46–49]. Those programmes were organised and delivered with a long-term perspective on how to reduce lifestyle risks to improve the health of the general population [50]. In addition, currently, many commercial enterprises have used the health promotion concept in their advertising efforts to promote the sale of their products. Therefore, the amount of available health promotion and disease prevention messages could be rather saturated in the public arena. Many of our study participants may have already come across this information before taking part in the study.

Nevertheless, the results from the current study revealed that the participants from the intervention group further advanced their CHD knowledge after the mHealth intervention, specifically, regarding the following points: 1) a family history of heart disease is a risk factor; 2) controlling blood glucose level helps reduce cardiac risk; 3) diabetic people tend to have low HDL but high cardiac risk; and 4) cardiac risk can be reduced by controlling one’s body weight within a normal range. These findings suggest that the mHealth and Care4Heart apps can be used as new and effective ways to supplement the existing programmes and services, provide disease prevention education to the public, and address some of the knowledge gaps. The revolution of mobile technology has changed how people share and consume data, from a passive receiver to a more active user. The Care4Heart app allowed users to obtain the right amount of information from the right sources and in a more accessible and convenient way, therefore enhancing their understanding and information retention. Furthermore, sending a daily SMS with healthy tips may have also contributed to some of the success of the intervention. An SMS is the least sophisticated and yet the most commonly used tool for transmitting simple information to end users [51]. It has been well established that SMS facilitate patient-provider communication and medication adherence as well as patient education and motivation [51].

In this study, differences were not found between the intervention group and the control group regarding the knowledge of other CHD risk factors, which included fatty foods, cholesterol level, exercise, gender, and smoking. This reflected the need for further app content modifications to better attract the users’ attention and support the uptake of additional information.

Our study indicated that there was no statistically significant difference in the perceived stress level between the two groups after the intervention. However, the participants in the intervention group had a lower mean PSS-10 score compared to their baseline, while the participants in the control group had a higher mean PSS score compared to their baseline. This may reflect a certain degree of usefulness for the relaxation exercise and stress management content included in the Care4Heart app in lowering the participants’ stress level. Nonetheless, four weeks may be too short to observe any changes in their coping style. Therefore, a longer study period should be considered to further validate such an effect. There were no significant differences in cardiac-related lifestyle behaviours between the two groups after the intervention, except for cholesterol control behaviour. Both groups seemed to improve their health screening behaviours regarding blood pressure and blood cholesterol during the four-week study period. This could be due to the increased awareness of CHD by taking part in the study, therefore leading to more screening behaviours at the beginning of the study. Both groups deteriorated in several health behaviours (e.g., regular exercise and alcohol consumption) after four weeks, but this was more pronounced in the control group. The percentage of participants from the intervention group that were exercising decreased from 62.5% (*n* = 25) to 60% (*n* = 24) while those from control group decreased from 77.5% (*n* = 33) to 65% (*n* = 26). In contrast to the health screening behaviours, which require relatively less individual effort, disease prevention behaviours that involve a change of lifestyle habits are much more difficult to implement. It requires more personal effort and self-discipline. Butland et al. [52] suggested that an effective behavioural change is normally assisted by a synergy of various interventions that are delivered over a sufficient period of time with a corresponding modification to the targeted variables. For this reason, the 4-week study period was rather short for lifestyle behavioural change and therefore was not sufficient to initiate other behavioural changes for the participants in both groups. Additionally, the content of our Care4Heart app was information-based, and information alone is not enough to bring about behavioural changes. A more interactive feature with real-time monitoring, sharing (socially), and feedback will be preferable in the future design of the mobile app, as this could increase the users’ self-awareness of their behaviour. For example, a patient’s physical activity levels can be assessed by an accelerometer or a heart rate monitor that is synchronised to their mobile phones. Patients can upload their training and physical activity data weekly and receive feedback from their healthcare providers [53] and their friends. The emergence of a social presence in the mobile app could help prompt behavioural changes.

In addition to the current primary management programmes of the SHF and the HPB, the SBCHDP programme provided a new and potentially effective way to engage people and increase their knowledge regarding CHD and its prevention. It permits more accessible opportunities for self-directed learning and relearning. The growing popularity of mHealth is evident because it promotes better acceptability and higher treatment adherence [28, 29]. It also has the potential to disseminate health information related to primary and secondary disease prevention to a larger population at a lower manpower cost. In the long run, with increased awareness and knowledge of CHD and its prevention, one hopes that more working adults will practise healthy behaviours and maintain a better health status. Consequently, with a healthier workforce, work productivity will increase.

### Limitations

There are some limitations to address. The use of convenient sampling limits the generalisability of the findings. A small sample size might have resulted in a reduction of power for the detection of significant differences. A short follow-up period restricted the evaluation of long-term effects. App technical failure in which the app crashed occasionally on certain old model smartphones and some old operating systems may have affected user experience during the course of the study. This problem was rectified by the study team soon after they received feedback from the participants, and it did not cause any major disruptions. However, we acknowledge that it may have affected certain participants’ access to the study intervention, which may have affected the participants’ engagement, leading to non-significant findings in certain measured variables. Monetary incentives might have caused sample selection bias, but it lowered the dropout rate. Lastly, it was challenging to monitor the participants’ intervention compliance.

## Conclusion

This pilot RCT has partially demonstrated the effects of a 4-week SBCHDP programme in improving awareness and knowledge of CHD as well as changing cholesterol control behaviour among the working population in Singapore. This study contributed some evidence to the mHealth research field and further affirmed that chronic disease prevention via mobile devices is feasible and effective due to its convenience. It represents a convenient, affordable, and accessible method for a substantial proportion of the population. In addition, our findings suggest that the SBCHDP programme can be provided to occupational health teams of different organisations as an educational programme for the prevention of CHD.

## Additional file

Additional file 1: Behavioural Risk Factor Surveillance System (BRFSS) Questionnaire. (DOC 62 kb)

## Abbreviations

BMI: Body mass index
BRFSS: Behavioural risk factor surveillance system
CHD: Coronary heart disease
CVD: Cardiovascular disease
HDFG-2: Heart disease fact questionnaires-2
HDL: High-density lipoprotein
ICC: Intraclass correlation coefficient
IRB: Internal review board
mHealth: Mobile health
MOH: Ministry of health
PSS-10: Perceived stress scale-10
RCT: Randomized controlled trial
SBCHDP: Smartphone-based coronary heart disease prevention
SHF: Singapore heart foundation
SMS: Short message service
