# Supplementary material for: The effect of a smartphone-based coronary heart disease prevention (SBCHDP) programme on awareness and knowledge of CHD, stress, and cardiac-related lifestyle behaviours among the working population in Singapore: a pilot randomised controlled trial
Source: Health Qual Life Outcomes. 2017 Mar 14;15:49. doi: 10.1186/s12955-017-0623-y (PMC5348869; doi:10.1186/s12955-017-0623-y)
Supplement: Additional file 1: — Behavioural Risk Factor Surveillance System (BRFSS) Questionnaire. (DOC 62 kb) [file 12955_2017_623_MOESM1_ESM.doc]

**Appendix**

**Behavioural Risk Factor Surveillance System (BRFSS) Questionnaire**

| **Personal Health Status**  Height_________ Weight_________  1. Which of the following groups does your age fit?   1. 21-34 years 2. 35-44 years 3. 45-54 years 4. 55-65 years | |
| --- | --- |
| 2. Would you say that your general health is:  a. Excellent  b. Very good  c. Good  d. Fair  e. Poor | |
| 3. Do you have a history of the following illness (*Please circle all the responses that apply to you*) | |
| 1. Diabetes 2. Cancer 3. Mental Health problem 4. Hypertension | 1. Stroke 2. Hypothyroidism 3. Respiratory illness |
| 4. Are you taking any prescription medication?   1. Yes 2. No   If yes, please specify | |
| **Weight** | |
| 5. How would you assess your own weight?  a. underweight  b. Acceptable or healthy  c. Overweight | |
| 6. What means do you use to assess your weight? (circling 1 answer only)   1. Comparison to others 2. Clothing size 3. Clothing fit (loose or tight) 4. Body mass index scales 5. How you feel 6. Opinion of others 7. Other (*Please State*) | |
| **Blood Pressure** | |
| 7. Have you had your blood pressure checked in the last 12 months?   1. Yes 2. No | |
| 8. Have you ever been told by a doctor, nurse or other health professional that you have high blood pressure?   1. Yes 2. No | |
| 9. Are you currently taking medication for your high blood pressure?   1. Yes 2. No | |
| **Exercise** | |
| 10. During the past month, other than your regular job, did you participate in any physical activities or exercise such as running, swimming, callisthenics, golf, gardening, or walking for exercise?   1. Yes 2. No (Skip Questions 58-60)   11. What type of physical activity or exercise did you spend the most time doing during past month?  a. please specific:______________  12. How many times per week or per month did you take part in this activity during the past month?  a. please specify: ______________/week (OR) ___________/month  b. I don’t know/ Not sure  13. And when you took part in this activity, for how many minutes or hours did you usually keep at it?   1. Please specify: _____________/ minutes (OR)____________hour (s) 2. I don’t know/ Not sure | |
| **Cholesterol** | |
| Blood cholesterol is a fatty substance found in the blood. | |
| 14. Have you EVER had your cholesterol checked?   1. Yes [go to question 62] 2. No [go to question 64] | |
| 15. How long has it been since you last had your blood cholesterol checked?   1. Within the past year (anytime less than 12 months ago) 2. Within the past 2 years (1 year but less than 2 years) 3. Within the past 5 years (2 years but less than 5 years ago) 4. 5 or more years ago | |
| 16. Have you EVER been told by a doctor, nurse or other health professional that your blood cholesterol is high?   1. Yes 2. No | |
| **Tobacco Use** | |
| 17. Have you smoked at least 100 cigarettes (5 packs) in your entire life?   1. Yes [go to question 65] 2. No [go to question 67] | |
| 18. Do you now smoke cigarettes every day, some days or not at all?   1. Every day 2. Some days 3. Not at all [go to question 67 ] | |
| 19. During the past 12 months, have you stopped smoking for one day or longer because you were trying to quit smoking?   1. Yes 2. No | |
| **Alcohol Consumption**  In Singapore, one standard drink is equal to 220 ml of beer, 100 ml of wine (about 1 small glass) or 30 ml of liquor (about 1 shot glass).  Examples of standard drink quantities:   | Beer | 1 can of beer = 1.33 standard drinks  1 bottle of beer (750 ml) = 3.4 standard drinks | | --- | --- | | Wine | 1 bottle (750ml) = 7.5 standard drinks | |  | 1 cask (4 litres ) = 40 standard drinks | | Other drinks | 1 stubby of cider (375ml) = 1.5 standard drinks  1 bottle of spirits (750ml) = 25 standard drinks | | |
| 20. During the past 30 days, have you had a least one drink of any alcoholic beverage such as beer, wine, a malt beverage or liquor?   1. Yes [go to question 68] 2. I drink, but no drinks in past 30 days [go to question 69] 3. Never drink | |
| 21. How often do you have a drink containing alcohol?  a. I don’t drink alcohol   1. Monthly or less 2. 2 to 4 times a month 3. 2 to 3 times a week 4. 4 or more times a week | |
| 22. How many standard drinks do you have on a typical day when you are drinking?   1. 1 or 2 2. 3 or 4 3. 5 or 6 4. 7 to 9 5. 10 or more | |
| 23. How often do you have 4 or more drinks on one occasion?   1. Never 2. Less than once a month 3. Once a month 4. Weekly 5. Daily or almost daily | |
